# Retinoic Acid: Sexually Dimorphic, Anti-Insulin and Concentration-Dependent Effects on Energy

**DOI:** 10.3390/nu14081553

**Published:** 2022-04-08

**Authors:** Joseph L. Napoli

**Affiliations:** Graduate Program in Metabolic Biology, Department of Nutritional Sciences and Toxicology, The University of California-Berkeley, Berkeley, CA 94704, USA; jna@berkeley.edu

**Keywords:** adiposity, androgen, dimorphism, estrogen, glucagon, insulin, liver, muscle, pancreas, retinoic acid

## Abstract

This review addresses the fasting vs. re-feeding effects of retinoic acid (RA) biosynthesis and functions, and sexually dimorphic RA actions. It also discusses other understudied topics essential for understanding RA activities—especially interactions with energy-balance-regulating hormones, including insulin and glucagon, and sex hormones. This report will introduce RA homeostasis and hormesis to provide context. Essential context also will encompass RA effects on adiposity, muscle function and pancreatic islet development and maintenance. These comments provide background for explaining interactions among insulin, glucagon and cortisol with RA homeostasis and function. One aim would clarify the often apparent RA contradictions related to pancreagenesis vs. pancreas hormone functions. The discussion also will explore the adverse effects of RA on estrogen action, in contrast to the enhancing effects of estrogen on RA action, the adverse effects of androgens on RA receptors, and the RA induction of androgen biosynthesis.

## 1. Introduction

All-trans-retinoic acid (RA), one biologically active vitamin A (retinol) metabolite, “wears many hats”. Conception, embryogenesis, energy balance, control of adiposity, neurological function, pancreatic development and islet cell maintenance, cellular immunity, and spermatogenesis all depend on RA [1,2,3,4,5,6]. This review will focus on the fluctuations and actions of RA with changes in energy balance and RA’s sexually dimorphic actions. The evaluation of these two aspects of retinoids have begun recently. A novel observation has revealed an RA increase with fasting and a decrease with re-feeding [7]. This enables RA to optimally exert its anti-insulin actions during fasting, and to decrease its interference with insulin actions during re-feeding. The former optimizes RA action and the latter optimizes insulin action. A brief discussion of the retinoid metabolon will provide context for the main focus. In addition, discussions of retinoid toxicity, actions in diabesity, and the impact of retinoids on pancreatic development and islet maintenance will provide further context to evaluate the literature addressing RA/insulin interactions. Much effort has gone into understanding the contributions of RA to embryonic development. Comparatively, limited insights address functional differences between the sexes and the impact of retinoid effects during the development of post-natal metabolic diseases. Yet, diet and nutrient function during the perinatal period to influence postnatal health span and longevity [8].

## 2. The Retinoid Metabolon

### 2.1. RA Homeostasis

**Figure 1:**
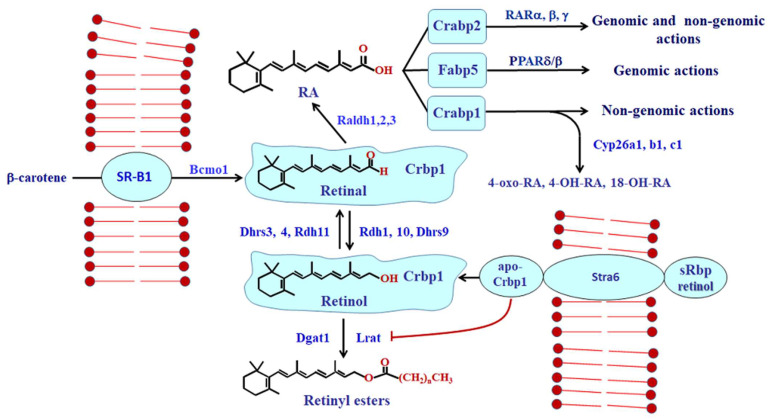
**Model of RA homeostasis.**

The retinoid metabolon includes multiple binding proteins, retinol dehydrogenases (Rdh), retinal reductases, retinal dehydrogenases (Raldh) and catabolic enzymes (Cyp) (Figure 1). Earlier reviews provided detailed accounts of these complex paths [9,10,11,12]. Briefly, the serum retinol-binding protein (sRbp) delivers retinol to cells through two membrane receptors. Stra6 serves as an extra-hepatic receptor for sRbp, whereas Rbpr2 (not shown in the figure) serves as a liver receptor. Cellular retinol-binding protein type 1 (Crbp1, encoded by *Rbp1*) recognizes the domain of Stra6 that extends into the cytoplasm and sequesters retinol inside cells with high affinity (*k*_d_ ~ 1 nM). The Crbp1-binding pocket buries the alcoholic group deep within, protected from the cellular milieu. Crbp1 releases retinol only upon interaction with enzymes that recognize both the binding protein and retinol. Holo-Crbp1 delivers retinol for esterification to lecithin:retinol acyl transferase (Lrat) through substrate channeling. The consecutive actions of Crbp1 and Lrat ensure one-way transfer of retinol from serum into the cell interior. Because apo-Crbp1 inhibits retinyl ester formation (inhibits Lrat) and holo-Crbp1 channels retinol to Lrat, the ratio apo-Crbp1/holo-Crbp1 directs retinol flux into and out of retinyl esters. As the concentration of apo-Crbp1 increases, the limited holo-Crbp1 directs retinol to RA biosynthesis by channeling retinol to Rdh. Rdh catalyze the rate-limiting reaction of the path from retinyl esters to RA [13].

Carotenoids delivered by the scavenger receptor-B1 (SR-B1) undergo central cleavage by β-carotene oxidase type 1 (Bco1) to produce retinal. Retinal reductases (Dhrs3, Dhrs4, Rdh11) reduce retinal into retinol. At least one reductase, Dhrs4, also accesses retinal bound with Crbp1; apo-Crbp1 inhibits this reductase [14]. This preserves sufficient retinal for RA biosynthesis. 

**Figure 2:**
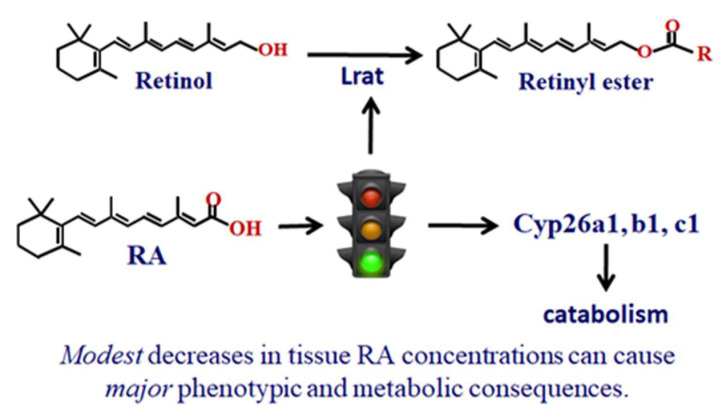
**RA regulates its concentrations by removing retinol into esters and inducing catabolic enzymes.**

Raldh1, 2, and 3, encoded by *Aldh1a1, 2, 3*, convert retinal irreversibly into RA. Crbp1 also directs retinal to retinal dehydrogenases. Although both Raldh1 and Raldh2 access retinal bound with Crbp1, each reacts differently to apo-Crbp1. Apo-Crbp1 inhibits Raldh1, but not Raldh2. Thus, the collective actions of holo-Crbp1, apo-Crbp1, Lrat and multiple Rdh, retinal reductases, and Raldh control retinol, retinal concentrations and RA biosynthesis. RA regulates its own concentrations by inducing Lrat and Cyp26A1, B1 and C1 (Figure 2). Three RA-binding proteins (Crabp1, Crabp2, Fabp5) deliver RA to specific nuclear receptors, and/or modulate non-genomic RA actions [15,16]. Crabp1 and 2 also channel RA to CYP for catabolism, thereby contributing to RA homeostasis [17,18,19,20]. Generally, the retinoid metabolon consists of an inter-related functional complex of enzymes and binding proteins.

None of these binding proteins or enzymes operates alone. Each depends on the presence of the entire metabolon for substrates and often for expression intensity. For example, Dhrs9 represses *Aldh1a1* mRNA; the loss of *Rdh1* results in decreases in *Cyp26b1* and increases in *Dhrs9* and *Aldh1a1* mRNA; partial ablation of *Rdh10* associates with increased *Dhrs9* expression in female muscles [13,21,22].

### 2.2. Complexity of Retinoid-Related Enzyme Actions

Cells co-express multiple Rdh, Raldh and Cyp26, both in overlapping and in different subcellular locations. Ablating one of these genes can induce compensation by others, but gene knockouts also reveal different phenotypes for each. These observations suggest redundant RA generation in some locations, yet production of distinct RA pools in others. For example, *Rdh1* ablation leads to enhanced adiposity in mice fed a low-fat diet, if the diet does not contain a copious amount of vitamin A [21]. *Rdh1* ablation causes obesity by disrupting RA adaptation to fasting and impairing brown adipose lipolysis and mitochondrial function, leading to a decreased body temperature, decreased oxidative phosphorylation, and enhanced lipid storage [23]. In contrast, the heterozygotic *Rdh10* knockout causes obesity only in mice fed a high-fat diet (HFD) [24]. Ablations of *Cyp26* and *Aldh1a* isoforms, respectively, also cause different phenotypes [25,26,27,28,29].

The *Rdh1* knockout confirms the fasting vs. re-feeding effects of retinoid actions. Three hundred and twenty-eight BAT (brown adipose tissue) genes differ after re-feeding in the KO compared to WT. Ninety-two genes differ during fasting. Only four genes differ in the KO from WT during both fasting and re-feeding. These data reinforce the importance of assaying RA, members of the retinoid metabolon and retinoid actions during either fasting or re-feeding, and avoiding the ad lib fed state.

A cautionary note: Rdh1 and Dhrs9 may be overlooked as important because of low mRNA expression. In fact, during the fed state, *Rdh1* mRNA occurs at very low or even undetectable levels (by qPCR) in liver (Section 8). However, its mRNA increases during fasting, and as detailed above, *Rdh1* ablation produces a prototypical retinoid-deficiency phenotype with respect to adiposity and mitochondria function. *Rdh1* ablation also reduces liver retinyl ester depletion (use of retinol for RA biosynthesis), and evokes increases in BAT *Aldh1a1* and *Dhrs9* mRNA, and decreases in *Cyp26b1* mRNA. Moreover, the intensity of mRNA expression does not necessarily reflect an enzyme’s metabolic contribution. A knockout serves as a more reliable metric. The *Rdh1* knockout provided unmistakable insights into its contribution to RA biosynthesis.

Another cautionary note: Raldh1 recognizes multiple substrates other than retinal and serves multiple functions. It functions as a retinal dehydrogenase with cellular specificity. In astrocytes and liver, Raldh1 converts retinal into RA [13,30,31]. In neurons, Raldh1 does not convert retinal into RA, but contributes to γ-aminobutyric acid biosynthesis [13,32]. Other substrates recognized by Raldh1 include aldehyde peroxidation products and oxazaphosphoranes. Raldh1 serves in the cornea and lens of mammalian eyes as an eta-crystalline and elsewhere as an androgen-binding protein. Raldh1 localizes to nuclei in addition to the cytoplasm, suggesting a gene regulating function. Clearly, Raldh1 has numerous functions other than retinal oxidation. The mere expression of Raldh1 cannot, therefore, designate a site of RA biosynthesis, nor can the phenotype of its knockout be assigned solely to disruption of retinal metabolism.

## 3. Vitamin A/Retinoic Acid Hormesis

### 3.1. RA Hormesis

**Figure 3:**
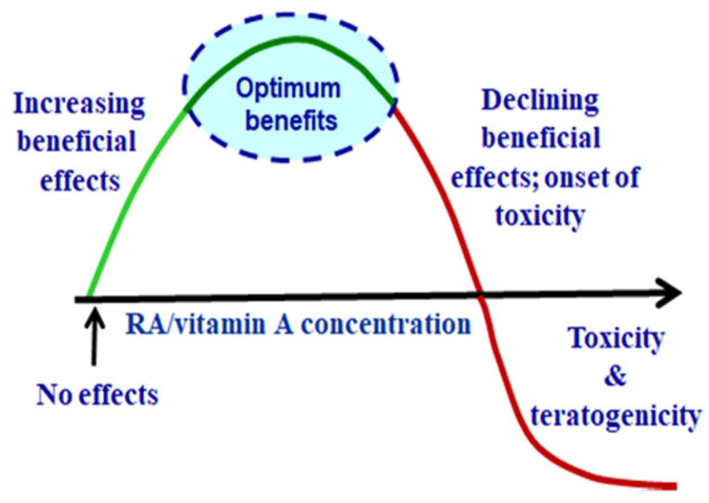
**Vitamin A and RA exhibit hormesis**. Retinoids display qualitative dose–response effects.

The pharmacological and toxic activities of vitamin A and RA do not necessarily reflect their physiological effects. Outcomes do not coincide because vitamin A and RA exhibit hormesis. Hormesis manifests as a dose–response curve in which lower concentrations produce beneficial effects, but higher concentrations produce divergent outcomes, including toxicity [33]. An inverted “J” or “U” shape denotes a dose–response curve that reflects hormesis (Figure 3). The species dosed also determines retinoid toxicity [34]. Monkeys and humans react with much more sensitivity to RA toxicity than rats or mice. The vehicle and route of administration influence toxicity, with oral dosing producing less effects than parenteral administration, and oil vehicle producing less effects than organic vehicles [35,36]. Physiologically reliable results require the judicious use of exogenous RA at lower doses and attention to the animal model, route of administration and formulation.

### 3.2. Contrary RA Effects

Weight variation as a function of vitamin A status offers an example of retinoid dose–response effects. Rats fed a vitamin-A-sufficient diet gain weight, grow and thrive. Rats fed a vitamin-A-deficient diet reach a weight plateau within weeks, and then lose weight and fail to thrive. They die within a few days after deficiency. Mammals fed insufficient vitamin A become obese. RA toxicity also manifests in anorexia and weight loss [37,38]. A dose (10 mg/kg) greater than the toxic amount (~3 mg/kg) does not cause weight loss in mice after 4 days, but a 100 mg/g dose causes a loss of ~4 g (~10% total body weight) in 4 days [39]. In the same experiment, the 10 mg/kg/day dose induced *Acc2*, but did not induce *Acox* or *Cpt1*, but a 50 mg/kg/day dose induced *Acot1* and *Cpt1*. In contrast, the physiological concentration of RA (~10–20 nM) ameliorated adiposity by restricting white preadipocyte (ST13 cell) differentiation [40].

Bone health and insulin release also react to retinoids in dose-dependent manners. A meta-analysis of epidemiological studies established a U-shaped relationship between serum retinol and hip fracture risk [41]. Both high- and low-serum retinol increase osteoporosis risk. An early study of vitamin A effects on insulin release in isolated rat islets provides another example of concentration-dependent vitamin A properties [42]. Additionally, 100 μM retinol, ~5000-fold more than the endogenous concentration in islets, inhibited both first- and second-phase insulin release, whereas 10 μM inhibited only second-phase release; 1 μM showed no effect. In stark contrast, 100 nM increased both phases of insulin release. Clearly, vitamin A and RA concentrations contribute to the effects that they produce!

## 4. RA Control of Preadipocyte Differentiation and Obesity

**Figure 4:**
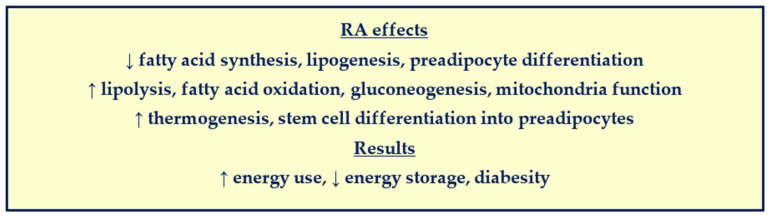
**RA regulation of energy balance**. Although RA supports pancreatic β cell development and maintenance, it also counters insulin actions, and thereby restricts diabesity. Arrows indicate increases or decreases.

A review covers RA actions in suppressing obesity, by opposing insulin action (promoting energy expenditure and inhibiting de novo lipogenesis) and inhibiting the differentiation of white preadipocytes into mature white adipocytes [43]. Briefly, Crabp2 delivers RA to RAR, which prevents insulin from down-regulating *Pref-1* transcription [44,45]. RA also promotes transcription of the metalloprotease TNFα converting enzyme (TACE), which cleaves a soluble peptide from Pref-1. The soluble peptide derived from Pref-1 retards the formation of mature white adipocytes [46]. Pref-1, along with TACE and the RA-induced transcription factor Klf2, induce Sox9 to inhibit the transcription of C/EBPβ and γ, which induce C/EBPα and Pparγ. The latter two prompt differentiation of white preadipocytes. Figure 4 summarizes the effects of RA on energy balance. Other reviews cover different aspects of RA action, namely promoting vascularization and mesenchymal stem cell differentiation in preadipocytes [47,48].

A few reports have concluded that Raldh1 generates retinal as an autacoid that opposes the RA action of restraining adiposity, and that low RA concentrations stimulate pre-adipocyte differentiation [49,50]. The majority of the literature indicates that RA inhibits preadipocyte differentiation at concentrations as low as 1 nM, well below its endogenous concentrations [24,51]. The reports that concluded retinal acts on its own relied on unskilled RA and retinal quantification, which produced values inconsistent with the literature. These reports also lacked crucial experiments. The contrary reports also presumed that injected retinal would reach adipose without undergoing metabolism—an improbable outcome for an aldehyde. The conclusions also overlooked alternative Raldh1 substrates and the non-enzymatic activity of Raldh1. A re-assessment of the *Aldh1a1* knockout extended findings by revealing that both males and females resisted diet-induced obesity, but only during the first 12 weeks after weaning [31]. This observation provides an incisive glimpse into a mechanism of adolescent adiposity in both sexes. The revisited study found that concentrations of retinal and RA in white adipose do not correlate with the phenotype caused by *Aldh1a1* ablation, based on sex, diet, genetics or age. Embryonic fibroblasts from *Aldh1a1*-null mice resist differentiating into adipocytes, but retain the ability to generate RA from retinol as well as WT, and respond to an RA receptor pan-agonist by. Differentiating. RA concentrations are as low as 1 nM, ~10- to 20-fold lower than physiological, impair adipogenesis of embryonic fibroblasts and NIH3T3-L1 cells [24,51]. Thus, the totality of avialable data indicate that Raldh1 functions as a retinoid-independent promoter of adiposity in white adipose only during early life.

## 5. Retinoids Direct Pancreatic Islet Development in Mammals

### 5.1. Emergence of Pancreatic Buds

Pancreatic buds begin to emerge on E (embryo day) 11 in rats, and on E9 to 9.5 in mice [52,53]. At this early stage, islet cells that develop in the mouse harbor glucagon, whereas insulin positive cells emerge on E10.5, with a burst of β cell proliferation on E14.5. Prototypical islets emerge on E18.5 with β cells located in their centers [54].

### 5.2. Vitamin A Induces Pancreas Development

Vitamin A deficiency introduced during pregnancy produces rat pups with reduced glucose-stimulated insulin secretion and promotes glucose intolerance [55]. Perfused islets from these vitamin-A-depleted rats retarded biphasic insulin release. Rats repleted with RA (8 µg/g diet), however, had normal insulin release. In a similar experiment, pups born from rat dams were fed a vitamin-A-insufficient diet (0.25 mg/kg or ~0.8 IU/g) before and during pregnancy, and continued after weaning, had β cell numbers diminished by 50%, relative to dams fed a diet high in vitamin A (4 mg/kg or ~13.3 IU/g) [56]. No clear effects emerged with α-cells, in contrast to subsequent reports. The continuous feeding of a vitamin-A-insufficient diet to adult mice reduced plasma insulin levels by 55% and increased serum glucose by 76%, relative to rats fed a diet high in vitamin A (chow with 30 IU/g).

### 5.3. RA Induces Pancreagenesis

Knockouts of RA-biosynthesizing enzymes and *Rbp1* generated mechanistic insights into retinoid effects on pancreas development. Homozygous *Rdh10* ablation impairs pancreas development and causes embryonic lethality at E10.5 in C57BL/6 mice. In contrast, the pancreases from *Rdh10* heterozygous knockouts are reportedly “indistinguishable” from WT [57]. 

*Rdh10* expression occurs in the “pancreas region” on E9.0 and in the posterior foregut epithelium on E9.5, coinciding with Pdx1 immunofluorescence [57]. The surrounding mesenchyme, including the Raldh2-positive dorsal mesenchyme, also express *Rdh10*, indicating a source of retinal for conversion into RA. Homozygous *Rdh10* ablation prevents the formation of the dorsal mesenchyme and reduces the ventral pancreas. Endoderm-specific *Rdh10* deletion reduces the size of the dorsal pancreas and the density of early hormone-expressing cells in an RA-dependent manner. The model proposed has Rdh10 and Raldh3 generating RA in the dorsal mesenchyme for delivery to the early endocrine cell cluster on E9.5. By E15.5, acinar cells rely on Rdh10 and Raldh1 to biosynthesize RA for delivery to the endocrine cell cluster.

Complementary research revealed that *Aldh1a2* expression occurs in the dorso-lateral mesenchyme abutting the dorsal pancreatic bud at the onset of pancreas specification on E9 [58,59]. *Aldh1a2*-ablated mice do not express *Pdx1* in the dorsal pancreatic bud, but the ventral bud appears normal. The lack of *Aldh1a2*-expression associates with dorsal pancreatic agenesis, but not ventral pancreatic agenesis. Its location and partial impact suggests an indirect function of Raldh2, i.e., secreting RA as a paracrine autacoid to promote dorsal pancreatic evolution, nor do early glucagon-expressing cells develop in *Aldh1a2*-ablated mice. The pancreatic epithelium initiates *Aldh1a1* expression from E13.5, first preceding and then coinciding with the burst of β cell proliferation that starts on E14.5 [59]. This presence of Aldh1a1 could suggest a key function in generating RA for total pancreagenesis. The absence of a retinoid-related phenotype in the *Aldh1a1* knock out, however, and especially a lack of pancreatic agenesis and dysfunction, suggests either compensation by alternative routes of retinal production or a lack of Raldh1 function as a retinal dehydrogenase in pancreas [60]. A three-fold reduction in the ability of the *Aldh1a1*-null mouse to convert a 50 mg/kg oral dose of all-*trans*-retinol (~2 mg/mouse, i.e. toxic) into RA does not address a physiological contribution of Raldh1 to RA biosynthesis. Nevertheless, a contribution of RA was revealed in explants harvested from the E10.5 dorsal pancreas and cultured for six days in the absence or presence of 25 nM RA [59]. Without RA in the medium, mostly non-endocrine cells proliferated.

### 5.4. Requirement of Crbp1 for Normal Pancreagenesis

**Figure 5:**
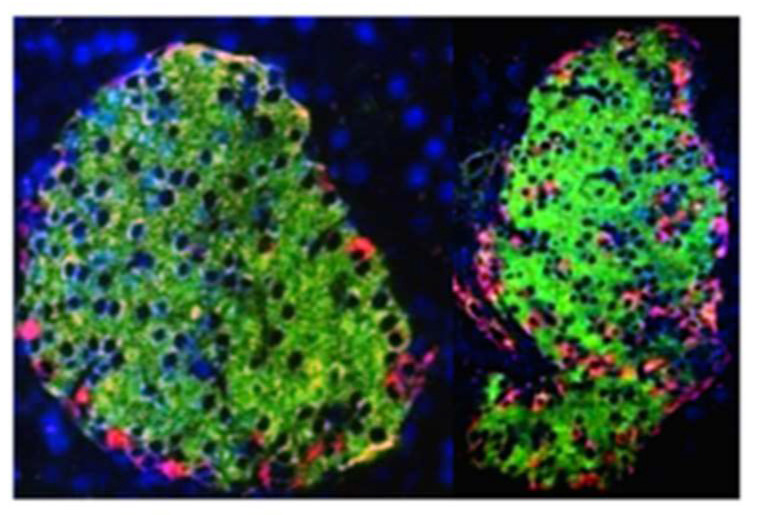
**Intrusion of pancreas α-cells into the β cell interior of mouse islets during vitamin A disruption**. Left, WT islets; right, *Rbp1*-null islets. Red indicates glucagon-secreting α cells. Green indicates insulin-secreting β cells. This phenomenon occurs in the Rbp1-null mouse and in the *Lrat*-null mouse [61,62].

*Rbp1* (encodes Crbp1) ablation decreases islet genes expression by 50% (*Pdx-1, Glut2, Gk*), which promote glucose sensing and insulin secretion. The islets in ablated mice exhibit α-cell infiltration into the β cell interior (Figure 5) [61]. *Rbp1*-null mice also have an AUC of the GTT (glucose tolerance test) ~2-fold greater than WT, whether fed a diet high in vitamin A (chow diet) or limited in vitamin A. Thus, Crbp1 ameliorates the high-vitamin-A effect of reducing glucose tolerance. Notably, diets high in vitamin A can reduce glucose tolerance, perhaps by limiting β cell function (see Section 5.5 and Section 5.6). Glucose intolerance and hyperglycemia in the *Rbp1*-ablated mouse are caused by hyperglucogonemia and hypoinsulinemia due to genetic and morphological alterations, which amplify gluconeogenesis. In contrast, excess dietary vitamin A does not diminish insulin sensitivity. *Rbp1*-null mice also experience increased fatty acid oxidation and resist obesity when fed an HFD. These data reveal that Crbp1 contributes to maintaining glucose homeostasis and energy metabolism through impacting pancreas endocrine cell type and function. Because these mice had *Rbp1*-ablated from conception, the impact of Crbp1 on pancreas development vs. the impaired function of a normally developed pancreas cannot be determined. Nevertheless, the data reveal the key function of Crbp1 in controlling retinoid homeostasis and physiological actions.

### 5.5. Retinoid Receptors and Pancreagenesis

Dominant-negative suppression of Rarα in all mouse *Pdx1*-expressing cells led to decreases in α, β and δ cells [63]. The same suppression, restricted to *Neurog3*-expressing endocrine progenitor cells, reduced the number of β cells on E16.5, without observable effects on the other endocrine cell types. The suppression of β cells resulted in a 25% increase in the AUC of an intraperitoneal GTT in 4- to 5-month-old mice, indicating at least a partial recovery of the β cell population. The H1 human embryonic stem cell line provided further insights into RA action. The pan-RAR antagonist AGN193108, added at the stage of *Neurog3* expression, decreased insulin RNA with no marked differences in glucagon or somatostatin RNA. Repression of RA signaling up-regulated Wnt signaling, suggesting that RA promotes β cell differentiation, but inhibits δ-cell transcription by antagonizing Wnt during the later stages of pancreas development.

### 5.6. RA and Human Pancreas Development

Findings for RA promotion of pancreagenesis were translated into human pancreas development [64]. A mutation (heterozygotic) in the transcription factor GATA6 causes pancreas agenesis in humans. RA induces expression of *GATA6*, which induces *GATA4*. This sequence stimulates development of pancreas progenitor cells and ensuing β cells in human pluripotent stem cells upstream of *PDX1*. Suboptimum RA partially impairs this process, but does not cause overt agenesis. Twenty-five nM RA markedly rescued *PDX1* expression in induced pluripotent stem cells generated from the GATA patient; although higher doses yielded a more complete rescue. The authors concluded that pancreas agenesis arises with the heterozygotic *GATA6* mutation only with severely repressed RA concentrations. Notably, the mouse *Gata6* heterologous mutation does not result in pancreas agenesis, illustrating major differences between humans and mice.

### 5.7. Summary RA and Pancreagenesis

Overall, RA provides essential signals to mouse, rat and human pancreas development with stage-specific actions. During early events, RA stimulates development of α, β and δ endocrine cells; during later stages, RA specifically promotes β cell differentiation, at expense of other endocrine cell types. Currently, protocols include RA in relatively low concentrations (100 nM) in the induction medium to generate insulin-producing β cells from human pluripotent stem cells ex vivo, as potential replacement therapy for diabetics [65].

## 6. Retinoids Maintain Pancreatic Islet Function in Post-Natal Mammals

### 6.1. Vitamin A Protects against Type 1 Diabetes

Vitamin A status affects postnatal pancreas function. Retinyl palmitate dosing to rats alleviated streptozotocin- and alloxan-induced diabetes (type 1 diabetes, T1D) [66]. When applied in vitro, retinyl palmitate had no effect, consistent with requiring metabolism into RA. RA dosing (2.5 mg/kg/d) to rats greatly reduced the streptozotocin impact on β cells [67]. High-dose formulations of RA and TGFβ in microparticles, given orally to C57BL/6 mice treated with streptozotocin also protected against T1D [68]. At 28 days after streptozotocin dosing, ~68% of the mice exposed to control microparticles experienced T1D, whereas only ~28% of those exposed to the RA and TGFβ-charged particles experienced T1D. In contrast, retinol reportedly worsened diabetes and insulitis (inflammation of the islets of Langerhans) in diabetes-prone BB/Wor rats fed a vitamin-A-deficient diet replete with retinol [69]. Perhaps these contrary observations reflect strain differences, or differences in the degree of depletion or the nature/amount of the dose. Nevertheless, humans with T1D have statistically significantly lower plasma concentrations of retinol than non-patients [70].

Autoreactive T cells cause T1D through pancreas β cell inflammation [71]. Because retinoids govern immunity, they might help restore peripheral tolerance mediated by dendritic cells that interact with T cells. Research with the NOD (non-obese diabetic) mouse corroborated the utility of pharmacological RA dosing for β cell protection after pancreas development. NOD mice develop spontaneous T1D during the post-natal period, similar to humans. Beginning as early as 3 weeks old, innate immune cells infiltrate NOD mice pancreata. Adding vitamin A to the diet (250 IU/g as retinyl acetate), >60-fold more than the 4 IU/g recommended by the National Research Council for rodents [72], starting at 3 to 5 weeks old, reduced the incidence of T1D from 71% to 25% in 7-month-old NOD mice [73]. Likewise, long-term pharmacological RA dosing (500 µg every other day/30 weeks) to pre-diabetic NOD mice with insulitis reduced T1D incidence 30% to 70% [74]. A second study concluded that long-term pharmacological RA treatment (20 mg/kg/d/3 weeks) for NOD mice prevented T1D at 13–19 weeks old [75]. Severe hyperglycemia resumed one week after terminating dosing. This work, establishing retinoid efficacy for protecting established β cells, seemed t to motivate recent work that applied sustained RA delivery to NOD mice. A formulation of microparticles containing RA and TGFβ1 allowed low-dose RA release (1 µg/day as tested in vitro with 10 mg of particles) along with TGFβ1 [76]. Particles were coated with the insulin B9-23 peptide to direct them to dendritic cells. When subcutaneously injected 5 times over the course of 21 days, the particles rendered NOD mice who were ~68% diabetes-free vs. 0% for controls at 120 days. The experimental mice remained diabetes free up to 33 weeks old. Because mice were not hyperglycemic at the age of injection (11 weeks), this formulation prevented the onset of T1D. Altogether, these reports indicate that post-development islet function requires continued RA presence, just as the RA requirement for islet development.

### 6.2. RA Inhibits Glucagon Secretion

RA (10 and 100 nM) inhibited glucagon secretion ~60% in intact rat islets in vitro [77]. In hamster-derived In-R1-G9 cells, both retinol (0.175 to 5.0 µM) and RA inhibited glucagon secretion. In the mouse αTC-1 cell line, retinol (1.75 μM) and RA (1 to 100 nM) inhibited glucagon secretion by as much as 80%. These observations support an RA requirement to maintain β cells in the post-natal period, while suppressing α cell proliferation. As noted above, α cell infiltration of the β cell interior occurs during disruption of retinoid homeostasis [61,62].

### 6.3. The Advantage of Avoiding Diets Copious in Vitamin A Content

Generally, mice born from dams fed a diet high in vitamin A (chow) do not become vitamin-A-deficient until 45 weeks old, even if fed a vitamin-A-deficient diet beginning at 4 weeks old [78]. Mice radically differ from rats in this regard [79,80]. Under the same conditions, rats become deficient in 6 to 10 weeks. In contrast, feeding a mouse colony a purified diet with 4 IU/g vitamin A (vitamin-A-sufficient) renders mice as susceptible to vitamin A deficiency as rats (after at least two generations fed the 4IU/g diet) [81]. The routine maintenance of a mouse colony fed a purified diet with the recommended vitamin A content has benefits. Chow diets have rescued or partially rescued knockouts of at least three vitamin A-related genes, *Rdh1*, *Rbp4*, and *Rbp2* [21,82,83]. Phenotypes emerged only after decreasing dietary vitamin A to the levels recommended for rodents by the National Research Council. Likely, other knockouts, such as *Crabp1* and *2*, with limited phenotypes, might display the same phenomenon. The point remains: chow diets have profound effects on vitamin A function relative to diets with sufficient but not high vitamin A content.

### 6.4. Pancreas Function in the Lrat-Null Mouse

Another approach to modulate tissue vitamin A involves *Lrat* knockout mice [84,85,86]. Homozygous *Lrat*-null mice fed a chow diet have greatly reduced liver retinyl esters (~15% of wild type) and suffer partial apermatogenesis and impaired rod and cone-supported visual functions. The continued presence of retinyl esters in liver and most tissues may reflect Dgat1 activity. (Dgat1 has retinyl ester biosynthesis activity and probably accounts for ARAT (acyl-CoA retinol ester transferase) activity) [87,88]. When fed a vitamin-A-deficient diet the *Lrat*-null mouse has severely depleted vitamin A tissue levels after 6 weeks, and therefore provides a model of vitamin A depletion.

Vitamin A insufficiency produced in the *Lrat*-null mouse resulted in decreased β cell mass due to apoptosis and associated hyperglycemia, indicated by impaired glucose tolerance in absence of insulin resistance [62]. Islets had α cell infiltration of the β cell inner mass, and an increase in α cell mass, resulting in hyperglucogonemia. A major reduction occurred in pancreatic Crbp1. The phenotype overlapped extensively with that of the *Rbp1*-null mouse in all similar experiments reported (aberrant GTT, normal ITT, hyperglucogonemia, α cell infiltration of the β cell inner mass), suggesting that the effects of vitamin A-depletion largely resulted from the decreased expression of *Rbp1* [61].

A dominant-negative *Rarα*-mutation controlled by a tamoxifen-inducible *Pdx1* promoter provided mechanistic insights into pancreas RA receptor function [89]. *Rarα* suppression decreased plasma glucose in mice and glucose-stimulated insulin secretion in isolated islets. As with the *Rbp1*-knockout, islet *Gk* and *Glut2* expression decreased, and *Rbp1* expression decreased by 70%. These mice did not experience glucose intolerance nor insulin resistance, but their islets had reduced insulin and increased glucagon. Thus, Rarα contributes to, but does not solely account for, the islet phenotype observed in *Rbp1*-null and *Lrat*-null mice. Dosing pharmacological amounts of the Rarβ2 agonists AC261066 and AC55649 (200 to 300 µg/day, 8 weeks) to ob/ob and db/db mice reduces the AUC of a GTT by ~20 to 38% and insulin resistance by ~50% [90]. The AUC of a GTT in mice fed an HFD decreasedt ~50% compared to untreated mice. Insulin tolerance improved to about the sensitivities of controls that were not exposed to diet-induced obesity. These data reinforce the insight that RA ameliorates Type 2 diabetes (T2D). In fact, in a clinical study of 60 patients, divided between those with poorly controlled T2D and healthy controls, the plasma levels of RA were ~1.4 ng/ml vs. ~1.8 ng/ml, respectively [91].

### 6.5. 9-cis-Retinoic Acid Reduces Glucose-Stimulated Insulin Secretion

An isomer of RA, 9-cis-RA (9cRA), occurs as an endogenous mouse pancreas autacoid, as established by liquid chromatography/tandem mass spectrometry [92]. Co-migration with an authentic standard in two hplc systems, followed by full-scan mass spectrometry, which verified its molecular weight and fragmentation pattern assignment, re-enforced the structural assignment [93]. If 9cRA occurs in other tissues, it would have concentrations <0.05 pmol/g. With a glucose load, 9cRA varies inversely and transiently. In pancreatic islets, 100 nM 9cRA transiently reduced glucose-stimulated insulin secretion; 9cRA functions by relatively and briefly reducing Glut2 and Gk activities. Ob/ob (lack leptin) mice had 2.2-fold higher 9cRA than WT. Glucose-intolerant db/db mice (lack the leptin receptor) also had higher 9cRA than controls. Mice with diet-induced obesity (DIO) had ∼2-fold higher 9cRA than controls. Only in pancreases from mice with DIO did an RA increase exceed the increase in 9cRA. Thus, β-cell-generated 9cRA operates as an autacoid in the pancreas. It exerts rapid local actions of brief durations. By attenuating insulin secretion and biosynthesis, and reducing glucose uptake into the pancreas, 9cRA prevents hypoglycemia. This allows short-term serum glucose, via glycogenolysis and gluconeogenesis, to increase to a greater extent than possible otherwise.

### 6.6. Summary of RA Effects on Post-Natal Pancreas Function

Overall, experiments with retinoid effects on post-natal pancreatic structure and function show that retinoids have physiologically and pharmacologically positive effects on preserving β cells and retarding α cell proliferation. These effects maintain a balance between glucagon and insulin secretion and protect against T1D and T2D. Notably, not all researchers agree that a clear association exists between retinol and RA vs. diabetes in humans [94]. This likely reflects difficulty in quantifying retinol and RA in serum and the wide range of normal blood retinol levels that defy characterization along simple lines of sufficiency vs. insufficiency.

## 7. The Serum Retinol-Binding Protein (Rbp4) and Insulin Resistance

The serum retinol-binding protein (sRBP, encoded by *RBP4*) transports lipophilic retinol through serum bound to transthyretin, and delivers retinol to tissues. sRBP binds with the receptor Stra6 (stimulated by RA 6), binding to its domains that project into blood [95]. Crbp1 recognizes Stra6 domains that project into the cytoplasm. This high affinity (k_d_ ~ 1 nM) retinol carrier acts as a sink that draws retinol into cells from sRBP through Stra6 [96,97]. Holo-Crbp1 associates with the retinol-esterifying enzyme Lrat to directly channel retinol to Lrat for esterification. The process assures unidirectional transport of retinol into cells without diffusion through the aqueous medium. Although Stra6 works bidirectionally in vitro, this conclusion only pertains to a specific protocol. Retinol will proceed down a concentration gradient from cells pre-loaded with retinol and devoid of Crbp1 and Lrat through Stra6, when such cells are presented with apo-sRBP. This does not duplicate the situation in vivo, where apo-RBP released from binding with Stra6 rapidly undergoes proteolysis, and esterification sequesters retinol inside cells.

sRBP4 correlates with insulin resistance in mice, obese humans, and in patients with T2D [98,99]. sRBP levels increase during insulin resistance. Its overexpression or dosing causes insulin resistance. The mechanism of insulin resistance presents an enigma because retinol delivery provides a substrate for RA biosynthesis, an anti-insulin autacoid. The mechanisms proposed indicate that sRBP binding to Stra6 initiates signaling that leads to insulin resistance [100,101]. The point remains that the mechanisms of sRBP and RA action do not relate directly to each other.

## 8. Insulin Suppresses Liver RA Concentrations

Because RA promotes pancreatic islet β cell proliferation and suppresses α cell proliferation, it might seem counterintuitive that RA would oppose actions of the β cell peptide hormone; insulin. yet instigate actions similar to those of the α cell peptide hormone glucagon. But it does. In contrast to insulin and similar to glucagon, RA intensifies energy use and opposes energy storage, through arresting pre-adipocyte differentiation in mature adipocytes, and promoting lipolysis, fatty acid oxidation, and gluconeogenesis. RA thereby suppresses insulin resistance and weight gain, in opposition to insulin effects [3,43,102,103]. The results with animal models pertain to humans. In a cross sectional study of 200 women, serum retinol and β-carotene levels were, on average, 1.3 and 61 µmol/L, respectively, in eutrophic subjects, in contrast to ≤1 and 44 µmol/L, respectively, for overweight or obese women [104]. A clinical study of men concluded that a suboptimal vitamin A status associates with increased adiposity in the 38% of the population who were genetically predisposed to obesity [105]. Based on these insights and the propensity of biology to evoke negative feedback (establish homeostasis), a reasonable question posed whether insulin counter-regulates RA biosynthesis or function. Such a counter action on RA would allow insulin to reach maximum impact during feeding and RA to achieve maximum impact during fasting.

**Figure 6:**
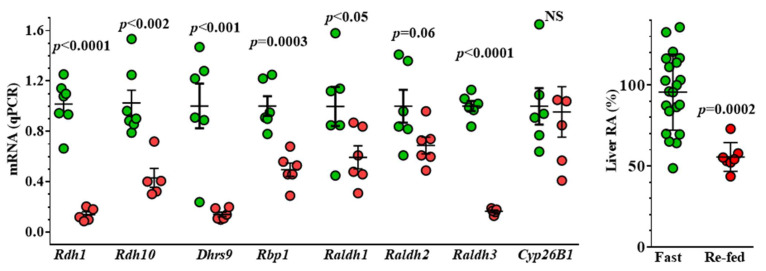
**Regulation of retinoid metabolon mRNA and RA concentrations in liver by fasting and re-feeding**. Mice were fasted for 16 h (green), or fasted for 16 h and re-fed for 6 h (red) for qPCR and re-fed for 9 h for RA quantification by LC/MS/MS. mRNA values represent data of 2–5 experiments, each with 4–8 mice per group and experiment. RA values show combined data from 3 experiments. In some experiments, re-feeding resulted in undetectable *Rdh1* mRNA.

Fasting increases expression of multiple enzymes and *Rbp1* in liver and increases RA (Figure 6). Note, however, that during feeding, liver *Rdh1* mRNA levels recede below detection (but not BAT *Rdh1* mRNA). Fasting also increases *Rdh1* and *Rdh10* mRNA in kidney. Mechanisms were evaluated in liver [7], because the liver systemically distributes macronutrients during feeding and produces fuels during fasting [106,107]. During feeding, to liver determines distribution of glucose to other organs and increases energy storage by generating glycogen and triacylglycerol. During to fasting, liver generates glucose and ketone bodies through gluconeogenesis, glycogenolysis and fatty acid oxidation, and in the long term, ketogenesis.

Timed re-feeding of mice fasted for 16 h reduced *Rdh1* and *Rdh10* mRNA in liver before reducing liver RA concentrations [7]. *Rdh1* and *Rdh10* mRNA decreased by 86 and 57%, respectively, in re-fed mice relative to fasted mice. The liver RA concentration decreased by ~41% 9 h after re-feeding. A glucose oral gavage had the same effect on both *Rdh1* and *Rdh10* mRNA, but glucose dosed by i.p. injection did not decrease *Rdh* mRNA. Oral glucose reaches the liver via the portal vein. This stimulus, combined with insulin, triggers an unidentified neuronal signal to increase net hepatic glucose uptake with a simultaneous impact on insulin action [108]. I.p. injection of glucose does not duplicate the physiological route of glucose uptake or neurological/hormonal responses to glucose uptake. Therefore, lack of an i.p. glucose effect establishes the physiological significance of Rdh down-regulation. Accordingly, injection of 0.5 IU/kg insulin to fasted mice achieved the same outcome.

Insulin, but neither glucose nor glucagon, decreases *RDH10* and *RDH16* (human homolog of mouse *Rdh1*) mRNAs in the human liver hepatoma cell line HepG2 [7]. Insulin, through the intermediacy of PI3K and Akt, excludes FoxO1 from the nucleus, which reduces *RDH* transcription. Insulin also increases the rate of *RDH* mRNA degradation. *RDH10* mRNA had a 35 h t½ in the absence of insulin. In the presence of insulin, the t½ decreased to 19 h. Inhibiting PI3K decreased the insulin destabilization of *RDH10* mRNA; whereas inhibiting Akt prevented it. A dominant-negative FoxO1 reduced *RDH* mRNA by ~80% and prevented the increase in RA biosynthesis from retinol.

In total, re-feeding, oral glucose, and insulin injection suppress *Rdh* mRNA and decrease RA in mouse liver. Insulin suppresses *Rdh* mRNA in HepG2 cells cultured in serum-free medium, through inhibiting transcription and destabilizing mRNA. PI3K and Akt inhibitors prevent insulin action. A dominant-negative FoxO1 construct decreases *Rdh* mRNA 40–80% relative to WT cells cultured in serum-free medium in absence of insulin. Glucagon had no effects.

The function of insulin in regulating Rdh expression was confirmed in the Akita mouse [109]. The Akita mouse harbors a mutation in the insulin 2 gene (Ins2+/C96Y), which results in abnormal processing of pro-insulin, damaging β cells [110]. Ultimately, the Akita mouse develops T1D. Five-month-old Akita mice lack insulin because of β cell loss and experience hyperglycemia and a decrease in body fat. *Rdh10* mRNA increased 2-fold in Akita mouse liver. Thus, these Akita mouse data reinforce the impact of insulin on *Rdh* mRNA and RA concentrations in liver, as first reported in [7]. Interestingly, *Cyp26a1* mRNA increased in liver of the Akita mouse. This may appear confusing because insulin absence leaves glucagon action unobstructed. If glucagon represses *Cyp26a1* mRNA (Section 9), why is there an increase? Because RA concentration increases in the absence of insulin. RA exercises a very potent effect on *Cyp26a1* induction; glucagon inhibits RA induction of *Cyp26a1* by only ~40%.

A difference in *Rdh* mRNA and RA did not manifest in a comparison of fasted mice to ad lib fed mice [109]. The protocol, however, essentially differed from the fasting/re-feeding protocol described in the report by Obrochta [7]. In the latter report, both groups of mice (fasting and re-fed) were starved for 16 h, and then the re-fed group was presented with food. With this protocol, re-fed mice rush to gobble up food when first made available. The re-fed protocol provides a relatively certain time of feeding onset and an explicit contrast between the energy-depleted and energy-sufficient states. During ad lib feeding (with no prior fasting), mice begin to eat at dark and finish at variable times, usually about midnight (~5 h later). At the time of euthanasia, ad lib fed mice do not synchronize in a fed state, especially during group housing. Such a variation reduces differences between fasting and ad lib fed states. Comparing fasted to ad lib fed mice may reveal only the most intensely expressed genes with the largest differences between the two states, and/or reflect a combination of partially fasted and fed mice. Relying on small numbers of mice per group (3 or 4) and not repeating experiments also “under powers” the data and reduces statistical significance. Given animal variation, basing metabolic conclusions on small numbers of mice in uncertain states of energy can foster false conclusions.

To summarize, insulin has the well-established action of excluding FoxO1 from the nucleus, resulting in FoxO1 proteolysis [111]. This eliminates transcriptional regulation by FoxO1, which in turn, eliminates *Rdh* induction and reduces *Rdh* mRNA stability, resulting in a decrease in RA.

## 9. Glucagon and Cortisol Diminish RA Induction of Liver *Cyp26a1*

**Figure 7:**
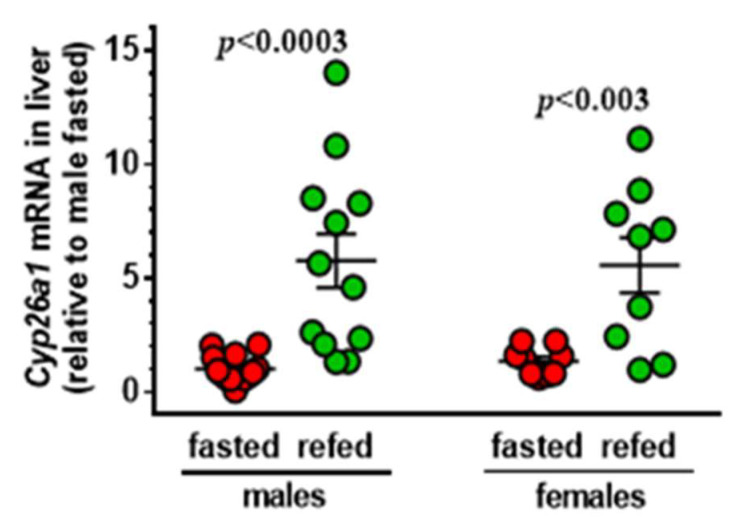
**Regulation of *Cyp26a1* mRNA in mouse liver by fasting and re-feeding**. Mice were fasted 16 h or re-fed 6 h after fasting for 16 h. Note the data spread in the refed mice. Using only 3 to 4 mice would underpower the data and could cluster data points toward one or the other extreme.

Three P450 enzymes specific for RA have been identified—CYP26A1, B1, C1—with A1 making the major contribution to RA catabolism in liver [27,29]. Liver intensely expresses Cyp26a1/CYP26A1 with more efficient kinetic constants than other P450s with RA activity: *K_m_* 9.4 nM; *V_max_* 11.3 pmol/min/mg protein [112]. RA vigorously induces CYP26A1 in HepG2 cells [113,114]. Because HepG2 cells had established insulin regulation of RA biosynthesis, my lab used them to determine the extent to which the regulation of *CYP26A1* contributed to the decrease in RA during feeding (Figure 7).

**Figure 8:**
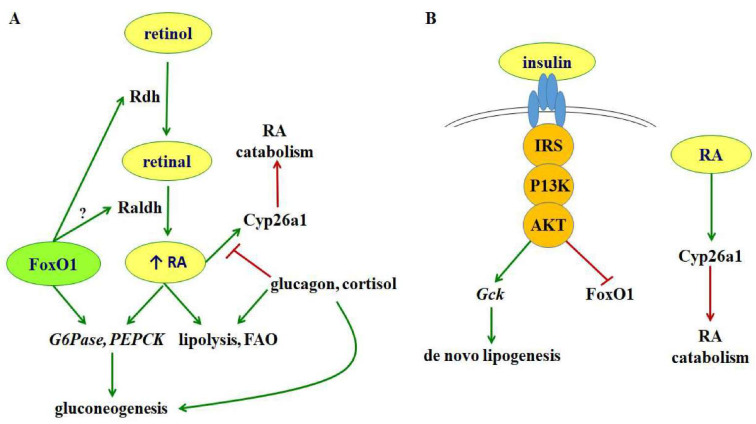
**Model of regulating RA homeostasis during fasting and re-feeding**. (**A**) During fasting, nuclear FoxO1 activates *Rdh* transcriptionally. Glucagon and cortisol restrain RA induction of *Cyp26a1*. FoxO1, RA, glucagon and cortisol stimulate gluconeogenesis. (**B**) After re-feeding, the increase in insulin expels FoxO1 from the nucleus; RA induces *Cyp26a1* unconstrained by glucagon and cortisol.

Fasting decreases and re-feeding increases *Cyp26a1* mRNA in mice (Figure 8), consistent with the hypothesis that insulin induces this gene [115]. Contrary to our initial hypothesis, although re-feeding increased *Cyp26a1* mRNA in mouse liver, neither glucose nor insulin had an effect. Instead, glucagon and cortisol, two hormones secreted during fasting, limited ability of RA to increase *CYP26A1* mRNA and reduced its catabolic activity in HepG2 cells. Glucagon and cortisol effects were neither additive nor synergistic, suggesting a common mechanism. Dexamethasone (Dex) duplicated cortisol inhibition. The glucocorticoid receptor (GR) antagonist, RU486, prevents the Dex effect on RA induction of *CYP26A1* mRNA. Dex did not repress CYP26A1 mRNA in the absence of RA induction. This indicates that the Dex-GR complex binds to the RA-RAR-RXR transcription co-activation complex to restrict transcription.

Interaction between C/EBPβ and the major allele of SNP rs2068888 enhances CYP26A1 expression; the minor allele restricts the C/EBPβ effect on CYP26A1. The major allele has been linked to increased acute coronary syndrome risk in the AIM-HIGH (Atherothrombosis Intervention in Metabolic Syndrome with Low HDL/High Triglycerides and Impact on Global Health Outcomes) trial [116]. In contrast, a GWAS study associated the minor allele (adenine) with a reduction of 2.28 mg/dL in blood triglycerides [117]. This suggests a link between the energy regulation of CYP26A1 and human health, because SNP rs2068888 occurs 2000 base pairs downstream of *CYP26A1.* CYP26A1 is the nearest annotated gene to the SNP. ChIP-seq assays show that the CCAAT-enhancer-binding protein beta (C/EBPβ) binds to the SNP in HepG2 cells [118]. RegulomeDB predicts the SNP as “likely to affect binding” to C/EBPβ [119]. siRNA knockdown of *C/EBPB* reduces its mRNA 50% in HepG2 cells, which decreases RA-induced *CYP26A1* mRNA 46 to 67%. RA recruits C/EBPβ to the SNP, but Dex enriches C/EBPβ in RARE1 of the *CYP26A1* promoter. These data indicate not only that the Dex-GR complex reduces RA induction of *CYP26A1* by binding to an RA-liganded coactivation complex of RAR-RXR, but it also reduces C/EBPβ-enhanced *CYP26A1* transcription. This accounts for the inter-related effects of CYP26A1 and the alleles of SNP206888 on blood triglycerides and human health. These effects stem from the ability of RA to diminish VLDL secretion from liver [39].

## 10. RA Exerts Tissue-Specific, Sexually Dimorphic Actions over Energy Use

RA exerts well-established pharmacological and/or toxic effects on adiposity, energy use and adipogenesis [120]. In contrast, insight has not kept pace into the post-natal effects of endogenous RA. Two drawbacks limit insights into endogenous RA effects. Hormesis creates an uncertain association between physiological effects vs. effects produced by exogenous RA dosing in higher amounts and/or feeding chow diets. The latter contain copious (much more than necessary and recommended) vitamin A. A second limitation involves inability to limit RA tissue concentrations in model animals reproducibly. Fortunately, ablating retinoid metabolon enzyme and binding protein expression by genetic intervention has allowed reproducible retinoid alterations in post-natal mice. Ablations that cause embryonic lethality can be pushed past the day of lethality to parturition by retinoid dosing [121]. In other cases, use of heterozygotes allows for survival, while sufficiently reducing RA to examine consequences.

The homozygous *Rdh10* knockout mouse dies at no later than E12.5 with multiple defects, including aspermatogenesis, dysfunctional pancreas development, and neurological and limb malformations [57,121,122,123,124,125]. On the other hand, the heterozygote (R10Het) lives and reproduces, permitting postnatal assessment of endogenous RA function [22,24]. RA concentrations decreased ≤25% in R10Het male and female livers and white fat depots. In gastrocnemius muscle, RA decreased by 38% and 17% in R10Het males and females, respectively (*p* < 0.02). Female R10Het experienced an increase in estrogen, stimulating a 1.7-fold increase in muscle mRNA of the retinol dehydrogenase Dhrs9 relative to WT because estrogen induces *Dhrs9* [126]. Increased *Dhrs9* in female KO relative to male KO contributed to the difference in muscle RA, as did a 33% decrease in female R10Het *Rdh10* vs. a 53% decrease in the male R10Het (*p* < 0.03). R10Het produced at least two unexpected outcomes. First, R10Het experienced modest decreases in liver RA that caused major changes in energy metabolism, adiposity, glucose tolerance and insulin resistance. Second, the effects were sexually dimorphic.

Male and female R10Het mice fed a HFD weighed >5 g and ~4.7 g, respectively, more than their WT littermates. Increases in fat caused body weight increases with no sizeable differences in lean body mass. The respiratory exchange ratio (RER, CO_2_ exhaled/O_2_ used) revealed that R10Het males experienced *decreased* fatty acid oxidation during the 12 h dark (feeding) cycle, but did not differ from WT during the light cycle. R10Het females experienced *increased* fatty acid oxidation throughout the entire 24 h, relative to WT. R10Het males displayed glucose intolerance and insulin resistance, but females experienced neither. R10Het males developed hepatosteatosis. Seventy-four percent of R10Het males had increased fat accumulation in liver with an average 2-fold increase in triglyceride, compared to 39% of WT. Females fed the HFD did not differ in liver triglyceride from WT. R10Het males, treated for 9 weeks with time-released low-dose RA, weighed 9 g less than the placebo-treated males and had body weights equivalent to mice fed an LFD. RA dosing also improved glucose tolerance and insulin sensitivity. The weight decrease occurred in fat, with no significant change in lean body mass. RA relieved hepatosteatosis and reduced the sizes of adipocytes in WAT. These data suggest the potential of chronic low-dose RA for treating obesity and associated metabolic disorders.

Bone (femurs) also reacted to *Rdh10* knock-down with sexual dimorphism. Adipocyte numbers increased 3-fold in female R10Het mice femurs. The increase in adipocytes clustered near the growth plate, the source of stem cells. Female femurs increased in length ~8.5% compared to WT. R10Het males, in contrast, experienced no significant differences in femur lengths or adipocyte numbers.

In run-to-exhaustion tests, WT males ran ~62 min, whereas R10Het males experienced a decrease of 40% in running time before exhaustion. WT females ran ~33 min (~47% less than WT males), but R10Het females ran ~72 min, ~2.2-fold longer than WT females and 10 min longer than WT males. Differences related to adaptations in gastrocnemius muscle RA. RA dissimilarities caused expression differences in multiple muscle genes. mRNA of the RA-regulated myogenic transcription factor *Myog* increased by ~2.8-fold in males, but decreased by 42% in females. *Myog*-null mice experience increased running endurance from improved fuel metabolism [127]. *Myh7* mRNA, an indicator of type I fibers (slow twitch), increased ~3-fold in R10Het male muscle. Type I fibers harbor abundant mitochondria and promote endurance through fatty acid oxidation. This increase in type I fibers proved futile, however, because the R10Het male mitochondria suffered gross impairment in the electron transport chain (cytochrome C oxidase), lipolysis (Hsl), glycogen storage (Gys), gluconeogenesis (Pck1, G6pc) and ATP biosynthesis. R10Het male muscles also contended with structural damage, indicated by increased centralized nuclei.

## 11. Sex Hormones Effect RA Concentrations and Action

The sexually dimorphic differences related to RA function likely stem from the dissimilarities in estrogen and androgen concentrations in males vs. females. Estrogen enhances RA signaling, and its biosynthesis, and promotes muscle function. Aside from inducing the Rdh *Dhrs9*, estrogen induces *RARβ* [128]. Estrogen also increases muscle mass and strength in vivo and promotes muscle regeneration [129]. Notably, estrogen intensifies muscle insulin sensitivity, prevents lipid accumulation and promotes metabolic health [130]. In contrast to positive estrogen effects on RA function, RA retards estrogen action [131,132]. The decreased RA in females, therefore, enhanced estrogen actions, which in turn limited the decrease in RA biosynthesis and signaling. In males, on the other hand, androgens retard RA function by repressing *Rara* and *Rarg* [133], whereas Rarb induces androgen biosynthesis [134].

## 12. Conclusions

New data have emerged indicating impact of fasting and re-feeding on RA function, sexually dimorphic RA actions, and developmental vs. post-differentiation RA effects.

To gain optimum insights into RA physiology:

- Experimental protocols should avoid chow diets with their higher (copious) amounts of vitamin A, which rescued several knockouts of the retinoid metabolon;
- RA dosing should heed hormesis effects;
- RA quantification should rely on fasted vs. re-fed states, and avoid ad lib fed animals;
- The assessment of RA-regulated gene expression, should consider fasting vs. re-feeding;
- Knockouts of retinoid metabolon genes to provide reproducible manipulation of tissue RA concentrations should be considered;
- Phenotypic evaluation should include both males and females;
- Experiments with model animals should require sufficient numbers to achieve reliable results.

## Data Availability

Data are contained within the article or within references.
